# Lethal chondrodysplasia in a family of Holstein cattle is associated with a *de novo* splice site variant of *COL2A1*

**DOI:** 10.1186/s12917-016-0739-z

**Published:** 2016-06-13

**Authors:** Jørgen S. Agerholm, Fiona Menzi, Fintan J. McEvoy, Vidhya Jagannathan, Cord Drögemüller

**Affiliations:** Department of Large Animal Sciences, Faculty of Health and Medical Sciences, University of Copenhagen, Dyrlægevej 68, Frederiksberg C, DK-1870 Denmark; Institute of Genetics, Vetsuisse Faculty, University of Bern, Bremgartenstrasse 109a, Bern, CH-3001 Switzerland; Department of Veterinary Clinical and Animal Sciences, Faculty of Health and Medical Sciences, University of Copenhagen, Dyrlægevej 16, Frederiksberg C, DK-1870 Denmark

**Keywords:** Congenital, Malformation, Rare disease, Type II collagenopathy

## Abstract

**Background:**

Lethal chondrodysplasia (bulldog syndrome) is a well-known congenital syndrome in cattle and occurs sporadically in many breeds. In 2015, it was noticed that about 12 % of the offspring of the phenotypically normal Danish Holstein sire VH Cadiz Captivo showed chondrodysplasia resembling previously reported bulldog calves. Pedigree analysis of affected calves did not display obvious inbreeding to a common ancestor, suggesting the causative allele was not a rare recessive. The normal phenotype of the sire suggested a dominant inheritance with incomplete penetrance or a mosaic mutation.

**Results:**

Three malformed calves were examined by necropsy, histopathology, radiology, and computed tomography scanning. These calves were morphologically similar and displayed severe disproportionate dwarfism and reduced body weight. The syndrome was characterized by shortening and compression of the body due to reduced length of the spine and the long bones of the limbs. The vicerocranium had severe dysplasia and palatoschisis. The bones had small irregular diaphyses and enlarged epiphyses consisting only of chondroid tissue.

The sire and a total of four affected half-sib offspring and their dams were genotyped with the BovineHD SNP array to map the defect in the genome. Significant genetic linkage was obtained for several regions of the bovine genome including chromosome 5 where whole genome sequencing of an affected calf revealed a *COL2A1* point mutation (g.32473300 G > A). This private sequence variant was predicted to affect splicing as it altered the conserved splice donor sequence GT at the 5’-end of *COL2A1* intron 36, which was changed to AT. All five available cases carried the mutant allele in heterozygous state and all five dams were homozygous wild type. The sire VH Cadiz Captivo was shown to be a gonadal and somatic mosaic as assessed by the presence of the mutant allele at levels of about 5 % in peripheral blood and 15 % in semen.

**Conclusions:**

The phenotypic and genetic findings are comparable to a previously reported *COL2A1* missense mutation underlying lethal chondrodysplasia in the offspring of a mosaic French Holstein sire (Igale Masc). The identified independent spontaneous splice site variant in *COL2A1* most likely caused chondrodysplasia and must have occurred during the early foetal development of the sire. This study provides a first example of a dominant *COL2A1* splice site variant as candidate causal mutation of a severe lethal chondrodysplasia phenotype. Germline mosaicism is a relatively frequent mechanism in the origin of genetic disorders and explains the prevalence of a certain fraction of affected offspring. Paternal dominant *de novo* mutations are a risk in cattle breeding, especially because the ratio of defective offspring may be very high and be associated with significant animal welfare problems.

**Electronic supplementary material:**

The online version of this article (doi:10.1186/s12917-016-0739-z) contains supplementary material, which is available to authorized users.

## Background

Chondrodysplasia is a developmental bone defect occurring due to disturbed endochondral osteogenesis. This leads to a reduced longitudinal growth of bones such as those of the limbs, spine and face. The most severe forms are found in lethal congenital generalized chondrodysplasia, which was originally reported in Dexter cattle in 1904 [1] and usually referred to as “bulldog calves.” Congenital chondrodysplasia has been described in a number of cattle breeds with varying phenotype and different modes of inheritance (OMIA 000004-9913, OMIA 000187-9913, OMIA 000311-9913, OMIA 000189-9913).

Development in molecular genetics has revolutionized the research in bovine teratology as the methods to identify genetic causes have improved significantly [2]. The molecular basis of a few bovine chondrodysplasias has been determined. In 2007, two independent mutations affecting the coding region of the aggrecan (*ACAN*) gene causing lethal chondrodysplasia in Dexter cattle (OMIA 001271-9913) were described. These *ACAN* mutations showed incomplete dominant inheritance, leading to a mild form of dwarfism in heterozygotes, while homozygous animals displayed extreme chondrodysplasia and usually died during gestation [3]. For another lethal chondrodysplasia phenotype (OMIA 001926-9913) reported in Holstein cattle in 2004 [4] it was later demonstrated that the sire (Igale Masc) of affected calves was mosaic for a dominant acting collagen type II (*COL2A1*) missense mutation (pG600D) disrupting the Gly-X-Y structural motif essential for the assembly of the collagen triple-helix [5]. In 2015, cases of stillborn bulldog-like calves occurring among the offspring of the Danish Holstein sire VH Cadiz Captivo (DK256588) were reported to the Danish bovine genetic disease programme [6]. This study reports the pathological investigation of the condition and the genetic analyses that led to the identification of a candidate causal mutation.

## Methods

### Animals

Three Holstein calves were submitted for examination: Case 1: a male delivered at gestation day (GD) 271; Case 2: a female delivered at GD 273; Case 3: a male delivered at GD 269. The cases originated from different herds. Ear tissue from two additional cases, where the herd veterinarian diagnosed the condition, was also submitted. The Holstein bull VH Cadiz Captivo was registered as the sire in all cases. Ethylenediaminetraacetic acid (EDTA) stabilized blood from all five dams was also available as was semen and EDTA stabilized blood of the sire.

### Post mortem examinations

Two calves (cases 2 and 3) underwent initial full body computed tomography (CT) scans using a single slice helical CT machine (Emotion, Siemens, Erlangen, Germany) to obtain a full view of the bone malformations. Slice thickness was 3 mm and surface rendered reconstructions, which help illustrate bone malformations, were made using OsiriX software [7]. The calves were then necropsied, which included longitudinal sectioning of the right limbs and the spine. Specimens of heart, lung, liver, kidneys, adrenal glands, thymus, vertebrae, femur and humerus were fixed in 10 % neutral buffered formalin for histology. The left limbs and the head of all calves were frozen at -20 °C and later examined by radiology. The head was sectioned longitudinally though the midline before radiology to obtain better images. The limbs were sectioned longitudinally after radiology to check for bilateral similarities in gross bone morphology. Tissues for histology were processed by routine methods, embedded in paraffin, sectioned at 2–3 μm and stained with haematoxylin and eosin. Bone specimens were decalcified in a 3.3 % formaldehyde/17 % formic acid solution before processing.

### Breeding analysis

Data on the outcomes of inseminations with semen of VH Cadiz Captivo in Denmark were obtained from the Danish Cattle Database. These included offspring recorded as “defective”, “stillborn”, “dead within 24 hours” or “still alive.” A questionnaire was constructed and mailed to owners of offspring recorded as “defective”, “stillborn” or “dead within 24 hours.” The questionnaire included pictures of two chondrodysplastic calves sired by VH Cadiz Captivo, the ear tack number of the dam of the calf and the date of delivery. The owners were then contacted by phone to assess if the offspring was chondrodysplastic or not.

Data on the gestation period for all calves sired by VH Cadiz Captivo were obtained from the database and the length of the gestation period for chondrodysplastic vs. normal calves was compared using the Welch Two-sample t-test.

### Genetic analysis

Genomic DNA was extracted for a total of 11 family members using standard protocols. For one of the affected calves the obtained quality of isolated DNA was very low due to tissue degradation. Therefore only a total of nine samples (four cases with their respective dams and VH Cadiz Captivo) were used for genotyping with the BovineHD BeadChip (Illumina), including 777,962 evenly distributed SNPs, at Geneseek.

MERLIN v 1.1.2 software [8] was used to analyze the dataset and carry out the linkage analysis. The Whittemore and Halpern non-parametric linkage pair statistics Z-mean and Kong and Cox logarithm of the odds (LOD) score were used to test for linkage using allele sharing among affected pedigree members without any assumptions on the mode of inheritance or on the genetic parameters of a specified model.

A polymerase chain reaction (PCR) free fragment library with a 390 base pair (bp) insert was prepared from one affected calf which was sequenced on half of a lane of an Illumina HiSeq3000 instrument using 2 × 150 bp paired-end reads. The genome sequencing data were deposited in the European Nucleotide Archive (ENA) (http://www.ebi.ac.uk/ena) under accession PRJEB12095. The mapping to the UMD3.1 bovine reference genome assembly and variant calling were undertaken as previously described [9]. During the mutation analysis, 118 sequenced genomes of normal cattle from 20 genetically diverse *Bos taurus* breeds were used as a local control cohort. The recent sequence variant database containing 1119 already sequenced genomes of the ongoing 1000 bull genomes project [5] was used as a global control cohort during filtering for private variants of the sequenced affected calf.

## Results

### Phenotype

**Fig. 1:**
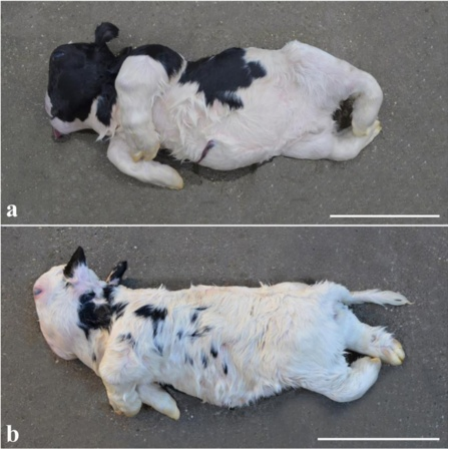
Gross morphology of chondrodysplastic calves. Notice the severe disproportionate dwarfism with short and compressed body and limbs and severe dysplasia of the facial bones. **a** case 1, **b** case 2. Bar = 30 cm

**Fig. 2:**
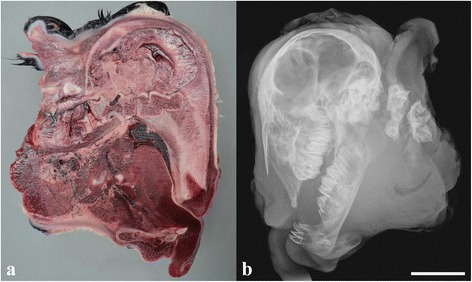
Lesions in the head of a chondrodysplastic calf. **a** Longitudinal sectioning of the head through the midline showing severe dysplasia and 90° angular ventral deviation of the facial bones to the axis of the brain (Rostral is to the right). The cerebellum appears compressed dorsally. Frozen specimen, case 1. **b** Radiograph of the head shown in “a” (Rostral is to the left). The splanchnocranium is dysplastic resulting in severe superior brachygnathism. Bar = 5 cm

The three submitted calves were morphologically similar and displayed severe disproportionate dwarfism (“bulldog” syndrome). The body and the limbs were shortened and compressed due to reduced length of the spine and the long bones of the limbs. The distal parts of the limbs were rotated medially and had digits of almost normal size. The limbs were bilateral symmetrically malformed. The face had severe dysplasia with compression and ventral deviation of the facial bones, which had an almost 90° angle to the axis of the brain stem. The dorsal aspect of the cerebellum was compressed (Figs. 1 and 2a). Palatoschisis was present in all cases and the tongue protruded. The body weight was reduced to 26 kg (25–28 kg) compared to full term Danish Holstein calves of approximately 43.5 kg for males and 41.5 kg for females.

Longitudinal sectioning of the spine and long bones of the limbs showed small irregular diaphyses of increased hardness and significantly enlarged epiphyses consisting entirely of a homogenous chondroid rubber-like tissue. Distinct epiphyseal lines could not be identified as the irregular metaphyses opposed the chondroid epiphyses that lacked a center of ossification. The dorsally enlarged epiphyses of the vertebrae caused compression of the spinal cord.

The thorax and abdomen were of reduced volume and the enclosed organs were consequently closely opposed. The heart had biventricular myocardial hypertrophia and dilation of the right ventricle. Slight hydrothorax was present. The lungs had diffuse congenital atelectasis. The liver surface was irregular and the texture of the parenchyma increased. Males had bilateral abdominal cryptorchidism.

**Fig. 3:**
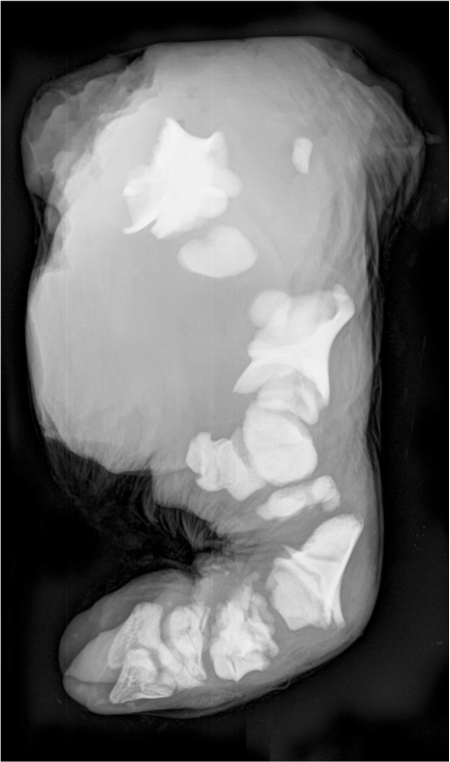
Radiograph showing abnormally developed bones of a hind limb. Only remnants of the diaphyses can be identified and the individual bones can only be identified by virtue of their relative location. The proximal bones are most deformed, while the distal phalanges are almost normal in shape and size. Case 2

Radiology and CT scanning showed that similar deformities were present in all calves. The neurocranium was relatively normal in size and shape (Fig. 2b). The splanchnocranium was foreshortened and misshapen resulting in severe superior brachygnathism. Mandibular incisors, premolars and molars were present. Maxillary teeth were also present but crowded due to the skull deformity. Bone structure appeared normal, with normal appearing cortical, cancellous and medullary cavities present in the mandible. Nasal and ethmoid turbinate bones were present. The appendicular skeleton was grossly deformed with the scapula, humerus radius, ulna, pelvis, femur, tibia, fibula and phalanges being affected. Only remnants of the diaphyses were identifiable. Bone morphology was so altered that they could only be identified by virtue of their relative location (Fig. 3). Deformities, especially shortening, were most severe for the proximal bones of the limbs while the distal phalanges were almost normal in shape and size. The carpus and tarsus were not ossified.

**Fig. 4:**
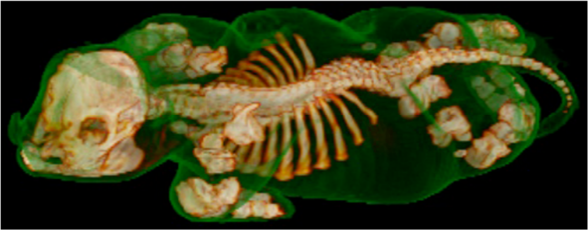
Surface rendered computed tomography images of a chondrodysplastic calf. The scanning data are rendered with bone and soft tissue surfaces. The latter has been set to have a degree of transparency in the reconstruction thus allowing visualisation of the calf’s overall morphology and its relation to the underlying skeletal abnormalities. Case 3

CT scanning showed lesions similar to those observed at radiology. In addition, there was widespread failure of fusion of the right and left parts of the dorsal spinous processes of the lumbar spine while partial fusion was present in the thoracic spine. Vertebral bodies were only partially formed in the cervical area and appeared as multiple osseous bodies, while vertebral bodies in the thoracic and lumbar spine were better defined but were nonetheless irregular in shape. Ribs (13 pairs) were present and appeared normal (Fig. 4). A movie showing the malformation in a 360° view is presented in Additional file 1.

**Fig. 5:**
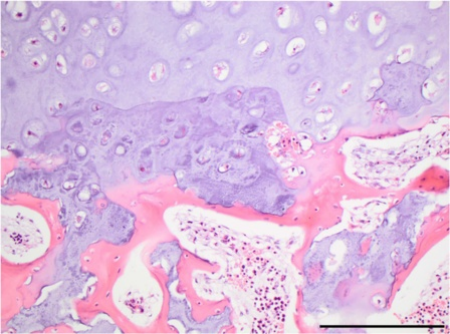
Photomicrograph of the growth zone. The growth zone is characterized by irregular areas a granular basophilic appearance and degeneration of chondrocytes. Notice absence of the alignment of chondrocytes normally seen in a growth zone. Large cores of chondroid matrix persists into the metaphysis. Vertebra, case 1, haematoxylin and eosin, bar = 200 μm

Histology showed highly irregular growth zones dominated by hypertrophied chondrocytes and with lack of normal alignment of chondrocytes although some chondrocytes were occasionally arranged normally in columns. Calcification of the intercellular matrix and chondrocyte degeneration occurred as irregular and interrupted areas towards the metaphysis. The ossification process was incomplete and cores of cartilage persisted throughout the meta- and diaphysis (Fig. 5). The epiphyses consisted of hyaline cartilage with chondrocytes located in variably sizes lacunae and with fibrous septae containing dilated vessels. Slight fibrosis and stasis was present in the liver.

### Breeding analysis

Analysis of breeding data showed that HV Cadiz Captivo had 521 recorded offspring of which 412 were born alive and had received an ear tack number, i.e. regarded as normal. The owners of the remaining 109 calves were contacted by questionnaire and phone. Sixty-four owners reported that the offspring had been a “bulldog calf” while 39 reported that the calf was of normal proportions. Six calves were excluded as the owner did not respond or could not recall the morphology. HV Cadiz Captivo therefore produced “bulldog calves” at a ratio of 12.4 % (64/515). Pedigree analysis of affected calves did not display obvious inbreeding to a common ancestor.

The length of the gestation period between cows having normal offspring *vs.*“bulldog calves” was compared. The mean among the cows with a normal calving was 280.2 days, which was significantly different (*P* < 0.0001) from the mean among cows giving birth to “bulldog calves” (276.2 days).

### Genetic analysis

**Fig. 6:**
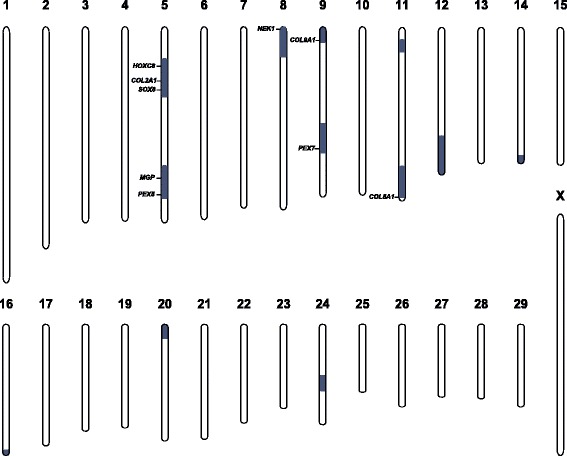
Non-parametric multipoint linkage analysis for chondrodysplasia. A total of 12 significantly linked genome regions are shown in blue. Note, that 9 out of 95 candidate genes for chondrodysplasia including *COL2A1* are located in linked regions

A multipoint non-parametric linkage analysis detected 12 genomic regions located on nine different chromosomes significantly linked with the chondrodysplasia phenotype at chromosome-wide error probabilities for Z-mean values and LOD scores below 0.05 (Fig. 6; Additional file 2). In light of the few reports of mutations causing chondrodysplasia in livestock, a causative variant affecting one of the known candidate genes was hypothesized. An OMIM database search for osteochondrodysplasia and/or chondrodysplasia revealed a total of 95 candidate genes (Additional file 3) of which nine genes were located in linked genome regions (Fig. 6).

**Fig. 7:**
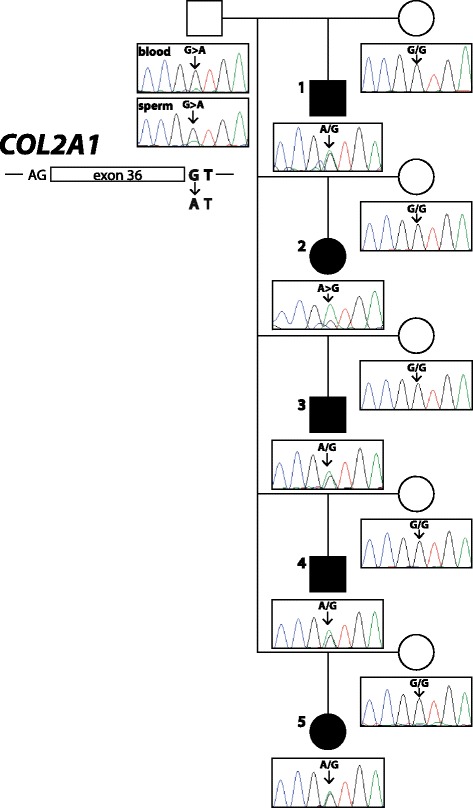
A *de novo* germline mutation in *COL2A1* causes chondrodysplasia. Genalogical chart showing five Holstein calves affected by chondrodysplasia (fully black symbols) and their parents. Males are represented by squares, females by circles. Note, that the electropherograms presented below the pedigree symbols show that the mutant A allele of the *COL2A1* intron 36 splice site mutation is present in heterozygous form in chondrodysplasia affected offspring and in the sire’s blood and semen at different ratios. The DNA of case 2 was of very low quality explaining the low ratio of the wild type G allele

For mutation analysis the whole genome of one affected calf was sequenced to 16.9x coverage using next-generation sequencing technology. Genome-wide filtering for sequence variants in the whole genome that were present only in the affected calf and absent from 118 control cattle genomes (that were sequenced before in the course of other on-going projects) resulted in 6054 private variants (Additional file 4). Subsequent filtering of variants located apart from the linked genome regions allowed the exclusion of 87 % variants remaining with 785 variants including 11 coding. Finally the candidate genes present in the mapped linked regions were screened for possible variants remaining with a single splice site variant in the *COL2A1* gene (Chr 5 g.32473300 G > A). All five available affected calves, their dams and the sire were genotyped by direct sequencing of a targeted PCR product using standard Sanger sequencing in the pedigree for this *COL2A1* variant revealing a perfect association of the mutant A allele with the chondrodysplasia phenotype (Fig. 7). All affected animals were heterozygous for the mutant allele and none of the dams carried this allele. Interestingly, sequencing showed the presence of the identified mutation in the sire VH Cadiz Captivo, who clearly carried the mutant allele at a low level in comparison to the wild type allele. The presence of both the wild type G allele and a smaller sized peak for the mutant A allele was detected in both available samples. The manually estimated relative area ratio of the mutant A allele was about 5 % in peripheral blood and about 15 % in semen (Fig. 7).

## Discussion

The gross, microscopic and radiographic lesions in the examined offspring of the sire VH Cadiz Captivo are consistent with generalized congenital chondrodysplasia, a condition usually referred to as “bulldog calf syndrome.” Pedigree analysis of affected calves did not display obvious inbreeding to a common ancestor, suggesting the causative allele is not a rare recessive. The normal phenotypes of the sire and the dams suggest a dominant inheritance with incomplete penetrance or a mosaic mutation. Due to the lethal effect of the mutation we concentrated on variants that were located within the coding sequences or within the splice sites of the candidate genes in the identified linked region of the bovine genome. Finally, mutation analysis for chondrodysplasia detected a perfect association between the splice site mutation of *COL2A1* and the disease phenotype. The *COL2A1* variant (c.2463 + 1G > A) is predicted to affect splicing because it alters the conserved splice donor sequence GT at the 5’-end of intron 36, which was changed to AT. The predicted consequences are either skipping of exon 36 or retention of intron 36. Both possible scenarios will lead to aberrant splice variants encoding truncated proteins, but nonsense-mediated decay might be the most likely major consequence of the splice site mutation. Unfortunately, the RNA was too degraded by the time the calves were available for examination so reverse transcription PCR failed to verify the consequence of the sequence variant experimentally.

Heterozygous mutations of *COL2A1* (OMIM 120140) have been identified in human patients with various rare autosomal dominant conditions characterized by skeletal dysplasia, short stature, and sensorial defects collectively termed as type II collagenopathies [10, 11]. These disorders not only impair skeletal growth but also cause ocular and otolaryngological abnormalities. The classical phenotypes include the spondyloepiphyseal dysplasia (SED) spectrum with variable severity from lethal SED, including achondrogenesis type II and hypochondrogenesis (OMIM 200610), through congenital SED (OMIM 200160) to late-onset SED (OMIM 271700). One third of human *COL2A1* defects are dominant-negative mutations in the triple-helical region of alpha 1 (II) chains which disrupt the collagen triple helix [11]. These predominantly glycine to nonserine residue substitutions exclusively create more severe phenotypes like achondrogenesis type II and hypochondrogenesis and correspond to the bovine *COL2A1* allele causing chondrodysplasia in Holstein cattle [5]. In humans, splice-site mutations are assumed to cause haploinsufficiency through nonsense-mediated mRNA decay [10]. The *COL2A1* mutations resulting in a premature stop codon are found in less severe phenotypes such as Stickler dysplasia type I (OMIM 108300) characterized by ocular, auditory, skeletal, and orofacial abnormalities or Kniest dysplasia (OMIM 156550) characterized with short stature, restricted joint mobility, and blindness [10, 11]. Only in two cases, splice site mutations, similar to the mutation identified in this study, are shown to produce phenotypes at the lethal end of the type II collagenopathy spectrum [12, 13]. Therefore, it seems to be highly likely that the detected bovine *COL2A1* splice site variant explains the phenotype in the affected calves. Probably a functional haploinsufficiency of collagen II, resulting from degradation of mutant transcripts due to nonsense-mediated mRNA decay, leads to a quantitative deficit of structurally normal collagen II causing the major malformations observed in the affected calves.

This lethal syndrome was present in 12.4 % of VH Cadiz Captivo offspring and was associated with the *COL2A1* splice site variant being present in his germ cells, presumable in around 15 % corresponding to the prevalence of malformed offspring. Therefore this sire is confirmed as a founder mosaic as shown before for “Solid Gold” for ovine callipyge [14] and “Campus” for porcine myopathy [15]. On the other hand, the genetic analysis of blood from the sire showed that the mutant *COL2A1* allele was present in peripheral blood cells at a low level of about 5 %. These results reveal that VH Cadiz Captivo is a gonadal and somatic mosaic. Germline mosaicism is a relatively frequent mechanism in the origin of genetic disorders [16]. Depending on various factors, such as the gene involved and/or the degree of mosaicism, the carrier of somatic and germline mosaicism may be asymptomatic or may present with various symptoms. There are two possibilities for the existence of such a mosaicism: one is that the mutation occurs in a germ cell that continues to divide. The other possibility is that the mutation occurs very early in a somatic cell before the separation to germinal cells and is therefore present both in somatic and germinal cells [16]. This seems to be the most likely explanation for VH Cadiz Captivo as he carried the mutant allele in both somatic and germinal cells, although he appeared normal.

A similar chondrodysplastic phenotype was observed in the offspring of the Holstein sire Igale Masc but affected only 1–2 % of his offspring [4, 5], which indicates that the mutation probably occurred later in the development of the testicular germ cells than in VH Cadiz Captivo. These findings show that the proportion of malformed calves may vary considerably between offspring of sires carrying germ cell mutations.

Paternal dominant germ cell mutations is a risk in cattle breeding, especially because the ratio of defective offspring may be very high. Presence of a dominant mutation in a certain proportion of spermatozoa has been hypothesized in cases of osteogenesis imperfecta. Denholm and Cole [17] found this lethal bone disorder in 44 % of a clinically normal Friesian bull mated to unrelated females, while Agerholm et al. [18] diagnosed osteogenesis imperfecta in 9 % of the offspring of a normal Holstein sire. Paternal dominant germ cell mutations are a challenge to cattle breeding as diseased progeny may occur without inbreeding, which in other ways is the most significant challenge regarding bovine genetic diseases. Reporting systems and disease programs that allow proper diagnostic of defective calves and targeted genetic investigations are highly valuable to limit the occurrence of severe disorders as lethal chondrodysplasia and osteogenesis imperfecta. The sire VH Cadiz Captivo was omitted for breeding by the breeding association as soon as the condition was diagnosed in his offspring.

It is important to recognize that even within a single breed, cases of lethal generalized chondrodysplasia show allelic heterogeneity. The cases reported here had a morphology indistinguishable from the Igale Masc bulldog calves [4] even by detailed post mortem examinations. A mutation in the same gene was therefore possible to be the cause. However, lethal congenital chondrodysplasia in Holsteins has been reported previously. Although such cases may share the basic lesion, i.e. chondrodysplasia, during examination in the field, significant variation in the phenotype may be found during post mortem examination, e.g. the syndrome reported by Berger and Innes [19] had severe hydrocephalus, which distinguishes it from the *COL2A1* associated “Igale Masc” and “VH Cadiz Captivo” types. Investigations and reports detailing characteristics of chondrodysplasia phenotypes are needed.

## Conclusions

Genomic analyses identified a causative spontaneous dominant acting candidate causal mutation in *COL2A1*. This study provides a first example of a dominant *COL2A1* splice site variant associated with a severe lethal chondrodysplasia phenotype. This mutation must have occurred during the early development of the asymptomatic sire VH Cadiz Captivo. Germline mosaicism as a relatively frequent mechanism in the origin of genetic disorders explains the prevalence of a certain fraction of affected offspring. Paternal dominant *de novo* mutations are a risk in cattle breeding, especially because the ratio of defective offspring may be very high and be associated with significant animal welfare problems. It is therefore important to have surveillance programmes for congenital syndromes in cattle.

## Abbreviations

*ACAN,* aggrecan gene; bp, base pair; CT, computed tomography; EDTA, ethylenediaminetraacetic acid; ENA, European Nucleotide Archive; GD, gestation day; LOD, logarithm of the odds; PCR, polymerase chain reaction; SED, spondyloepiphyseal dysplasia

## Additional files

Additional file 1: Surface rendered computed tomography scanning movie of a chondrodysplastic calf. (MOV 2291 kb) Additional file 2: Non-parametric multipoint linkage analysis output data. Z-mean values and LOD scores and their chromosome-wide error probabilities (*P*) along a grid of equally spaced locations (2 Mb) of all 29 autosomes. The minimum and maximum achievable values in the present linkage analyses are given in the first two rows. Chromosome-wide significant Z-mean values and LOD scores and their *P*-values are highlighted in green. (XLSX 53 kb) Additional file 3: Candidate genes for chondrodysplasia. OMIM database search for genes associated with osteochondrodysplasia/chondrodysplasia and their genomic position in the cattle genome. (XLSX 17 kb) Additional file 4: Private sequence variants of the sequenced chondrodysplasia affected calf. A total of 6054 sequence variants which were absent in 118 control genomes. Variants (*n* = 785) located in the linked genome regions are highlighted in green. (XLSX 370 kb)

## References

[1] Seligmann MB (1904). Cretinism in calves. J Pathol Bacteriol.

[2] Nicholas FW, Hobbs M (2014). Mutation discovery for Mendelian traits in non-laboratory animals: a review of achievements up to 2012. Anim Genet.

[3] Cavanagh JA, Tammen I, Windsor PA, Bateman JF, Savarirayan R, Nicholas FW, Raadsma HW (2007). Bulldog dwarfism in Dexter cattle is caused by mutations in ACAN. Mamm Genome.

[4] Agerholm JS, Arnbjerg J, Andersen O (2004). Familial chondrodysplasia in Holstein calves. J Vet Diagn Invest.

[5] Daetwyler HD, Capitan A, Pausch H, Stothard P, van Binsbergen R, Brøndum RF (2014). Whole-genome sequencing of 234 bulls facilitates mapping of monogenic and complex traits in cattle. Nat Genet.

[6] Agerholm JS, Basse A, Christensen K (1993). Investigations on the occurrence of hereditary diseases in the Danish cattle population 1989-1991. Acta Vet Scand.

[7] Rosset A, Spadola L, Ratib O (2004). OsiriX: An open-source software for navigating in multidimensional DICOM images. J Digit Imaging.

[8] Abecasis GR, Cherny SS, Cookson WO, Cardon LR (2002). Merlin-rapid analysis of dense genetic maps using sparse gene flow trees. Nat Genet.

[9] Murgiano L, Shirokova V, Welle MM, Jagannathan V, Plattet P, Oevermann A (2015). Hairless streaks in cattle implicate TSR2 in early hair follicle formation. PLoS Genet.

[10] Nishimura G, Haga N, Kitoh H, Tanaka Y, Sonoda T, Kitamura M (2005). The phenotypic spectrum of *COL2A1* mutations. Hum Mutat.

[11] Barat-Houari M, Sarrabay G, Gatinois V, Fabre A, Dumont B, Genevieve D (2016). Mutation update for *COL2A1* gene variants associated with type II collagenopathies. Hum Mutat.

[12] Körkkö J, Cohn DH, Ala-Kokko L, Krakow D, Prockop DJ (2000). Widely distributed mutations in the *COL2A1* gene produce achondrogenesis type II/hypochondrogenesis. Am J Med Genet.

[13] Mortier GR, Weis M, Nuytinck L, King LM, Wilkin DJ, De Paepe A (2000). Report of five novel and one recurrent *COL2A1* mutations with analysis of genotype-phenotype correlation in patients with a lethal type II collagen disorder. J Med Genet.

[14] Smit M, Segers K, Carrascosa LG, Shay T, Baraldi F, Gyapay G (2003). Mosaicism of Solid Gold supports the causality of a noncoding A-to-G transition in the determinism of the callipyge phenotype. Genetics.

[15] Murgiano L, Tammen I, Harlizius B, Drögemüller C (2012). A de novo germline mutation in *MYH7* causes a progressive dominant myopathy in pigs. BMC Genet.

[16] Zlotogora J (1998). Germ line mosaicism. Hum Genet.

[17] Denholm LJ, Cole WG (1983). Heritable bone fragility, joint laxity and dysplastic dentin in Friesian calves: a bovine syndrome of osteogenesis imperfecta. Aust Vet J.

[18] Agerholm JS, Lund AM, Bloch B, Reibel J, Basse A, Arnbjerg J (1994). Osteogenesis imperfecta in Holstein-Friesian calves. Zentralbl Veterinarmed A.

[19] Berger J, Innes JR (1948). Bull-dog calves (chondrodystrophy, achondroplasia) in a Friesian herd. Vet Rec.

